# Cytochrome P450 26A1 modulates uterine dendritic cells in mice early pregnancy

**DOI:** 10.1111/jcmm.14423

**Published:** 2019-05-31

**Authors:** Ai‐Qin Gu, Dan‐Dan Li, Dan‐Ping Wei, Yan‐Qin Liu, Wen‐Heng Ji, Ying Yang, Han‐Yan Lin, Jing‐Pian Peng

**Affiliations:** ^1^ State Key Laboratory of Stem Cell and Reproductive Biology, Institute of Zoology Chinese Academy of Sciences Beijing China; ^2^ University of Chinese Academy of Sciences Beijing China

**Keywords:** CD86, CYP26A1, dendritic cells, ID2, implantation

## Abstract

Cytochrome P450 26A1 (CYP26A1) plays important roles in the mice peri‐implantation period. Inhibiting its expression or function leads to pregnancy failure. However, little is known about the underlying mechanisms involved, especially the relationship between CYP26A1 and immune cells. In this study, using *Cyp26a1*‐specific antisense morpholigos (*Cyp26a1*‐MO) knockdown mice model and *pCR3.1‐Cyp26a1* vaccine mice model, we found that the number of uterine CD45^+^CD11c^+^MHCII^lo‐hi^F4/80^−^ dendritic cells (DCs) was significantly decreased in the treated mice. The percentage of mature DCs (CD86^hi^) was obviously lower and the percentage of immature DCs (CD86^lo^) was remarkably higher in uterine DCs in the treatment group than that of the control group. Further experiments found that ID2, a transcription factor associated with DCs development, and CD86, a DC mature marker molecule, were both significantly reduced in mice uteri in the treated group. In vitro, ID2 and CD86 also decreased in bone marrow‐derived DCs under *Cyp26a1*‐MO treatment. These findings provide novel information that CYP26A1 might affect the embryo implantation via modulating the differentiation and maturation of uterine DCs.

## 1 INTRODUCTION

Cytochrome P450 26A1 (CYP26A1) is a member of the cytochrome P450 superfamily and participates in the degradation of retinoic acid (RA).^1^ RA is a derivative of vitamin A and has a wide range of biological effects. It has been reported that RA is a critical signalling molecule during vertebrate development. Strict regulation of its distribution in embryos is the key to normal morphogenesis.^2^ As a member of RA metabolic enzyme family, CYP26A1 has been confirmed to participate in protecting the development of certain embryonic tissues from inappropriate RA signalling.^3^ *Cyp26A1* knockout mice die during mid‐late gestation and display major morphogenetic defects, such as spina bifida, caudal and lumbosacral region truncation.^4^ CYP26A1 also has been confirmed that play pivotal roles in embryo implantation. Previous work in our laboratory found that the number of embryo implantation sites was significantly decreased after the uterine injection of *Cyp26a1*‐MO or anti‐CYP26A1 antibody.^5^ CYP26A1 has a specific temporal and spatial expression pattern in the mouse and rat uteri, which selectively expressed in lumen epithelium, glandular epithelium and decidua tissue and increased in the implantation phase.^5^, ^6^ Further work found that CYP26A1 regulated Th17 cells through *all‐trans*‐RA‐RARα signalling during the mice peri‐implantation.^7^ However, this pathway was proved to play a limited role in the recent work, which suggested that CYP26A1 might regulate NK cells through chemokines.^8^ It has been reported that dendritic cells (DCs) can regulate T‐cell‐mediated immune response^9^ and have a cross‐talk with NK cells at the foetal‐maternal interface.^10^ Are DCs involved in the CYP26A1 regulation of embryo implantation? There is no relevant report on this issue. It is necessary to explore whether CYP26A1 causing the pregnancy failure is related to DC.

Dendritic cells as the most powerful antigen‐presenting cell can capture and transfer information from the outside world to the cells of the adaptive immune system. They are critical not only for inducing primary immune response, but also for inducing immunological tolerance.^9^ DC with different phenotypes performs different functions. Immature DC displays low expression of major histocompatibility complex II (MHCII) and costimulatory molecules, whereas mature DC displays high levels of these molecules.^11^ It is generally believed that immature DC is mainly involved in recognition and phagocytosis of antigens and mediating immune tolerance; whereas mature DC, on the contrary, mainly participates in the antigen presentation process and mediating immune rejection.^12^ Pregnancy is a special situation in which the mothers coexist peacefully with hemi allogeneic foetuses. Many foetal, maternal and placental mechanisms work together to protect the foetes from immunological recognition and rejection.^13^ DC has been reported to play important roles in maintaining the normal course of pregnancy. Depletion of uterine DC leads to decidualization failure, implantation damage and embryo resorption.^10^, ^14^, ^15^, ^16^ Besides, adoptive transfer of DC could reduce the abortion rate of CBA/J × DBA/2J mice, which is a typical abortion‐prone model.^17^, ^18^ Recent work in our laboratory found that the balance between conventional DC and plasmacytogenic DC is pivotal for immunological tolerance during pregnancy in the mice.^19^ These reports indicate that uterine DCs have a critical function in the process of pregnancy. One of the most remarkable features of DCs at maternal‐foetal interface is its low density.^20^ But DC has strong antigen‐presenting ability, one mature DC can stimulate as many as 2000 T cells in vitro,^21^ so even a small amount of DCs in the decidua could be important at the maternal‐foetal interface.

In mice, uterine DCs have been well characterized by their expression of high levels of CD11c and MHCII surface markers without the expression of macrophage surface marker F4/80.^22^ CD86, also known as B7‐2, is a protein expressed by antigen‐presenting cells. It provides stimulatory signals for T‐cell activation and is often used to identify mature DCs.^23^, ^24^ In this study, we found that inhibition of CYP26A1 altered the numbers of uterine CD45^+^CD11c^+^MHCII^lo‐hi^F4/80^−^ DCs and their subsets (CD86^lo^ and CD86^hi^ DCs). To our knowledge, this is the first report that CYP26A1 may affect embryo implantation by regulation the uterine DCs.

## 2 MATERIALS AND METHODS

### 2.1 Mice

Eight‐ to ten‐week‐old healthy female and male BALB/c mice were purchased from Beijing Vital River Laboratory Animal Technology Co., Ltd. (Beijing, China). The mice were properly housed in the laboratory animal room of the Institute of Zoology, Chinese Academy of Science (Beijing, China), which is a temperature and humidity controlled room with a 12‐hr light/dark cycle. All the animal manipulation procedures were approved by the Institutional Animal Care and Use Committee of the Institute of Zoology, Chinese Academy of Sciences. We used syngeneic pregnant mice (BALB/c × BALB/c) in this study. Female and male BALB/c mice were caged at a 2:1 ratio for one night, and the appearance of a vaginal plug on the next morning was identified as gestational day 1 (GD1).

### 2.2 Morpholino antisense oligonucleotides knockdown mice

Morpholino antisense oligonucleotides (MOs) were used by intrauterine injection as described earlier with minor modifications.^5^, ^8^, ^25^ The following MOs were synthesized by Gene Tools, LLC (Philomath, OR): *Cyp26a1*‐MO (*Cyp26a1*‐specific antisense morpholigos), 5'‐CATGGCACGCTTCAGCCTCCCGCGC‐3'; and Std‐MO (standard control MO), 5'‐CCTCTTACCTCAGTTACAATTTATA‐3'. The MOs were dissolved in sterile ddH_2_O at a stock concentration of 4 mmol/L and stored in the 4°C refrigerator. 7.5 µL solution containing 30 nmol *Cyp26a1*‐MO or Std‐MO was injected into the uterine horn of each mouse on GD4, corresponding to the treatment group and the control group respectively. All the pregnant mice were sacrificed on GD7. The uteri were collected for further analyses.

### 2.3 Recombinant plasmid immunized mice

The construction and injection of recombinant plasmid was performed using the previous methods with tiny modifications.^5^, ^26^ Full‐length *Cyp26a1* cDNA was cloned from the pregnant rats uteri, using specific primers with *Hind*III/*Xho*I restriction sites (forward primer: 5'CGAAGCTT (*Hind*III) ATGGGGCTCCCGGCGCTGCT3'; reverse primer: 5'CGCTCGAG (*Xho*I) TCAGATATCTCCCTGGAAGTGG3'). The products were purified and cloned into *pGEM‐T* vector (Promega, Madison, WI). Then *pGEM‐T‐Cyp26a1* and the *pCR3.1* vector (Invitrogen, Eugene, OR) were cut by *Hind*III/*Xho*I (Promega) at 37°C for 2 hours. The recombinant plasmid *pCR3.1‐Cyp26a1* was constructed using T4 ligase (Promega) at 16°C overnight. Subsequently, the recombinant plasmid was digested by *Hind*III/*Xho*I at 37°C for 2 hours and the insert fragment was sequenced to confirm the accuracy of construction. The expression of the recombinant plasmid was detected according to the former methods with small modifications.^26^ Total 60 female mice were equally divided into two groups. One group was immunized with 80 µg *pCR3.1‐Cyp26a1* (dissolved in 100 µL saline) per mouse and regarded as the treatment group, and the other group was immunized with the same dose of empty *pCR3.1* per mouse as the control group. All the mice were immunized using thigh muscle injection. Twenty‐four hours before immunization, each mouse was injected with 100 µL of 0.25% bupivacaine at the same position as an adjuvant. Immunization was carried out every 7 days for a total of four times. On the third day after the last immunization, the female mice were mated with male mice at a ratio of 2:1. All the female mice were coupled with male mice in 3 weeks and sacrificed on GD6 or GD7. The uteri were obtained for further analyses.

### 2.4 Induction and culture of bone marrow derived dendritic cells

Bone marrow (BM) cells from BALB/c female mice were harvested from femurs and tibias as previously mentioned^27^ and cultured according to the method of another publication with minor modification.^23^ 2 × 10^6^ BM cells were seeded into 100 mm bacteriological petri dishes with 10 mL RPMI1640 (GIBCO BRL, Eggenstein, Germany) supplemented with 2 mmol/L glutamine, 100 U/ml penicillin (Sigma), 100 µg/mL streptomycin (Sigma), 50 µmol/L 2‐mercaptoethanol (Sigma), 10% FBS (Biolnd), and 200 ng rmGM‐CSF (Peprotech). After 3 days, another 10 mL of the same medium was added. At the sixth day, a half displacement method was used to replace the medium. The cells were collected and planted into 24‐well plate at day 7 with a confluence degree was about 70%‐90%. The fresh culture medium containing 5 nmol/L *Cyp26a1*‐MO was replaced in the next morning as the treated group. The control group was renewed by the fresh medium with 5 nmol/L Std‐MO. Cells were collected after two days for further analyses. All the cells were cultured with 5% CO_2_ in a humid incubator at 37°C.

### 2.5 Cell suspension preparation and flow cytometric analysis

Uterine tissue was dissected and minced into debris with scissors in HBSS solution containing 1 mg/mL collagenase type IV (Sigma‐Aldrich), 1 mg/mL bovine serum albumin (YEASEN, Shanghai, China) and 0.3 mg/mL hyaluronidase (Sigma‐Aldrich), and incubated at 37°C for 30 minutes, with minor modifications as previously described.^28^ After the digestion, cells were centrifuged to remove the supernatant and incubated in the PBS solution for 15 minutes at 37°C. Then the cell suspension was filtered through 400 lmnylon mesh. The single cell suspension was then prepared. After centrifugation, cells were resuspended in red blood cell lysis and washed with PBS solution. The cell suspensions were blocked with antimouse CD16/CD32 (14‐0161; eBioscience) at 4°C for 15 minutes and then incubated with fluorescently labelled antibody for half an hour. The following antibodies were used for flow cytometric analysis: antimouse CD45 PerCP‐Cyanine 5.5 (45‐0451; eBioscience), antimouse CD11c FITC (11‐0114; eBioscience), antimouse CD86 PE (12‐0862; eBioscience), antimouse F4/80 APC (17‐4801; eBioscience), antimouse MHCII BV421 (107631; Biolegend). Finally, the cells were washed and suspended with PBS solution for analysis on a FACSAria IIIu (BD Biosciences, Franklin Lakes, NJ) instrument. The percentages of uterine CD45^+^CD11c^+^MHCII^lo‐hi^F4/80^−^ DCs, immature CD86^lo^ DCs and mature CD86^hi^ DCs were analysed using FlowJo 7.6.1 software (Tree Star, Inc, Ashland, OR).

### 2.6 Single‐cell population transcriptome sequencing

Uterine CD45^+^CD11c^+^MHCII^lo‐hi^F4/80^−^CD86^lo^ immature DCs and CD45^+^CD11c^+^MHCII^lo‐hi^F4/80^−^CD86^hi^ mature DCs from the 7th day of normal pregnancy mice were sorted by FACSAria IIIu (BD Biosciences, Franklin Lakes, NJ) instrument. Cell precipitation was sent to Shanghai Majorbio Bio‐Pharm Technology Co., Ltd. (Shanghai, China) for RNA‐seq. Cells with good condition were selected for RNA extraction, amplification, construction of a DNA library and sequencing. Each step was strictly in accordance with transcriptome sequencing criteria.

### 2.7 Total RNA isolation and real‐time quantitative PCR

Total RNA was extracted from the uteri and marrow derived dendritic cells (BMDCs) with Trizol^®^ reagent (Invitrogen, Carlsbad, CA) and was reverse transcribed into stable cDNA using M‐MLV reverse transcriptase buffer (Invitrogen, Carlsbad, CA) in accordance with the manufacturer's instructions. The amplification of cDNA was performed using 2× UltraSYBR Mixture (CWBIO, Co. Ltd, Beijing, China). Real‐time quantitative PCR was carried out with a LightCycler 480 PCR instrument (Roche, Indianapolis, IN). The target gene mRNA expression was normalized to glyceraldehyde‐3‐phosphate dehydrogenase (GAPDH) expression. The fold change was calculated as 2^−ΔΔCt^ (cycle threshold). The primers used for quantitative PCR are listed in Table S1.

### 2.8 Western blotting

Mice uterine proteins were extracted using lysis buffer (Applygen, Beijing, China) with protease inhibitors (Roche). The concentration was determined by following the specifications of the Bicinchoninic Acid Protein Assay Kit (Pierce, Rockford, IL). Proteins were separated by 10% SDS‐PAGE and electrical transferred onto a nitrocellulose membrane (Pall, New York, NY). After being blocked in 5% skimmed milk powder made from TBST solution at room temperature for 3‐4 hours, the membranes were washed with TBST solution and incubated with primary antibodies at 4°C overnight. Rabbit source polyclonal anti‐CYP26A1 antibody (1:1000; ab151968, Abcam), anti‐Id2 antibody (1:500; PA5‐49683, invitrogen) and anti‐GAPDH antibody (1:1000; mAb #5174, CST) were used. Then the membrane were washed thoroughly in TBST solution and incubated with goat antirabbit IgG conjugated with horseradish peroxidase (HRP; KPL, Gaithersburg, MD) diluted by 1:10,000 in TBST at 25°C for 1 hour. Finally, the membrane was visualized and quantified on a Gene Gnome XRQ Chemiluminescence detector (Syngene, Cambridge, UK) using ECL Detection Kit (Pierce, Rockford, IL) and GeneSys software (VilberLourmat, France). The relative protein levels were analysed by Bio‐Rad Quantity One software (Bio‐Rad, Hercules, CA) and standardized to GAPDH.

### 2.9 Direct immunohistochemistry

The presence of anti‐CYP26A1 antibody in the mice uteri immunized with *pCR3.1‐Cyp26a1* recombinant plasmid was detected by direct immunohistochemistry according to the previous methods with some modifications.^26^, ^29^ The frozen uterine sections (7 µm) were washed in PBS solution and fixed in 4% paraformaldehyde for 15 minutes. Before incubated with 3% hydrogen peroxide, the sections were immersed in PBST solution (PBS with 0.03% Triton X‐100) for 15 minutes to permeate cell membranes. Sections were washed and blocked by 10% normal goat serum (ZSGB‐BIO, Beijing, China) at 37°C for 1 hour. Subsequently, the sections were incubated with an antimouse secondary antibody conjugated HRP (115‐035‐003, Jackson ImmunoResearch, USA) at 37°C for 1 hour. Then the nuclei were stained with haematoxylin (Sigma‐Aldrich, St. Louis, MO) and coloured by taking advantage of diaminobenzidine tetrahydrochloride detection kit (ZSGB‐BIO). Finally, the sections were washed with deionized water, dehydrated in ethanol gradient solutions, sealed with neutral resin, and captured with Nikon Eclipse Ni‐U microscope and NIS software (Nikon, Tokyo, Japan).

### 2.10 Statistical analysis

Data analysis was performed with GraphPad Prism 5.01 software (GraphPad Software, San Diego, CA). The results were shown as mean ± SEM using unpaired *t* test to evaluate the differences. Statistical significant was established when *P* < 0.05.

## 3 RESULTS

### 3.1 Dynamic changes of DCs in normal pregnant uteri during early pregnancy

**Figure 1:**
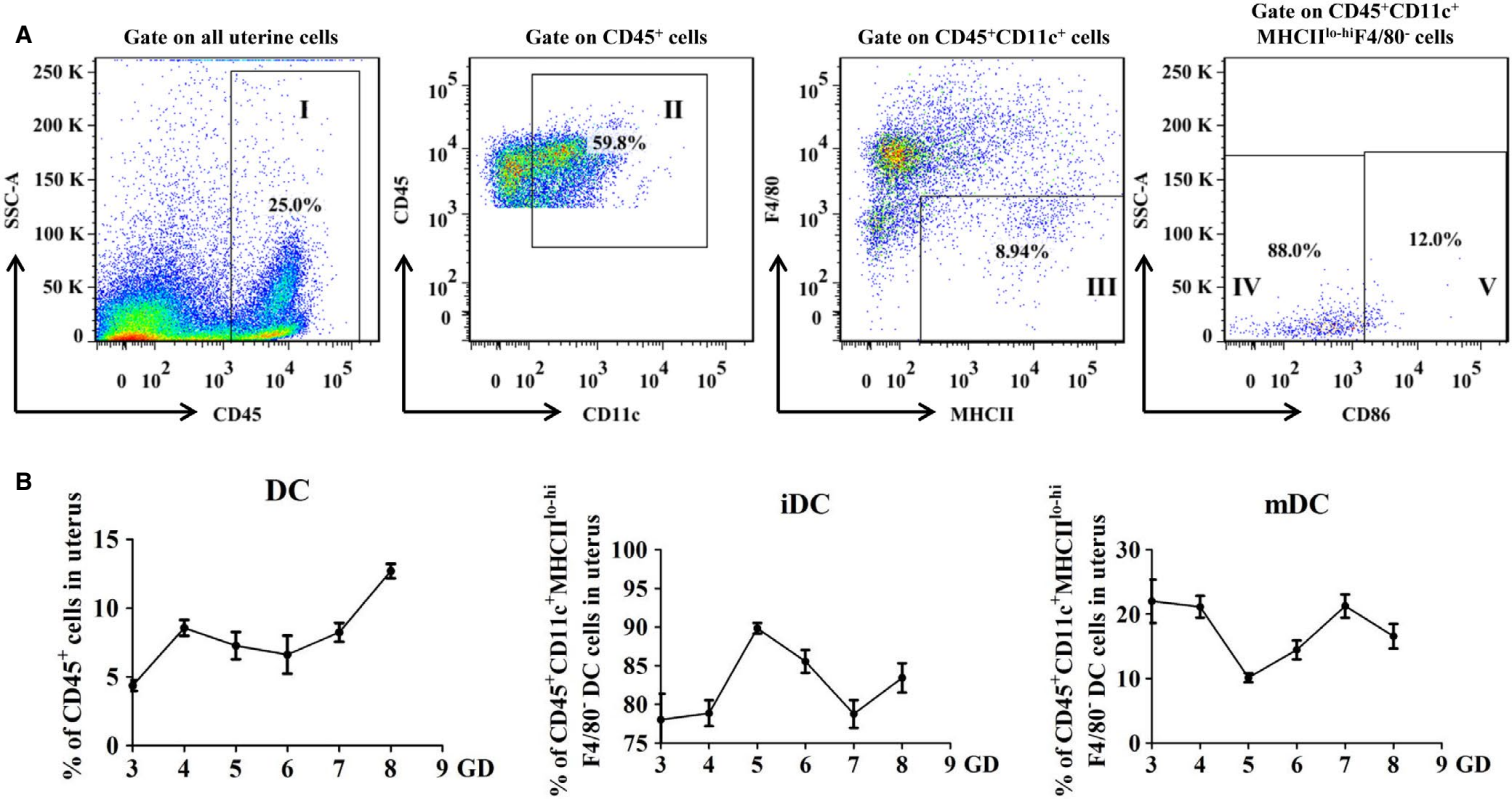
Flow cytometry analysis of the dynamic changes of uterine dendritic cells in mice during early normal pregnancy. (A), A representative result with detailed flow gate strategy. Gate I represents CD45‐positive cells which is to separate out immune cells. Gate II and III display CD11c^+^F4/80^−^MHCII^lo‐hi^ cells which have been considered as uterine DC cells. Gate IV displays the immature dendritic cells with low expression of CD86; Gate V displays the mature dendritic cells with high expression of CD86. (B), Respectively displayed the percentage of uterine DCs in mouse uterine immune cells and the percentages of immature and mature DCs in mouse uterine dendritic cells. D3, D4, D5, D6, D7, D8 represented, respectively, the third, fourth, fifth, sixth, seventh, eighth days of pregnancy. At least three independent biological repeats were counted at each time point. DC, dendritic cell; iDC, immature dendritic cell; mDC, mature dendritic cell

In order to explore whether CYP26A1 participating in embryo implantation is related to the percentage of uterine DCs, it is necessary to establish an undisturbed pattern to analyse the dynamic changes of uterine DCs in the early pregnancy. In this study, the percentages of uterine DCs (uDCs) in the immune cells and their subsets immature and mature DCs in the uDCs from the normal mice early pregnant uteri were analysed by flow cytometry. As shown in Figure 1, we used CD45^+^ to identify the immune cells and used CD11c^+^MHCII^lo‐hi^F4/80^−^ to identify uDCs. The immature and mature DCs were further identified by CD86^lo^ and CD86^hi^ respectively. The detailed gate strategy was shown in Figure 1A. The percentage of uDCs in mice displayed to rise on the fourth day of pregnancy, and maintained a relatively constant level during the peri‐implantation period (from gestation day 4 to 7), then significantly increased on the eighth day (Figure 1B). Among these uDCs, more than 70% were immature DCs, whereas mature DCs accounted for less than 30%. Immature DCs (iDCs) exhibited an upward‐downward‐upward fluctuation pattern during the early pregnancy. The proportion of iDCs in uterine DCs reached the highest level on GD5 and the lowest level on GD7. While mature DCs (mDCs) presented just on the contrary. Uterine mDCs decreased from GD3 to GD5, and increased from GD5 to GD7, then declined from GD7 to GD8 (Figure 1B).

### 3.2 Cyp26a1‐MO knockdown mice significantly impacted the percentages of uterine DCs and their subsets

**Figure 2:**
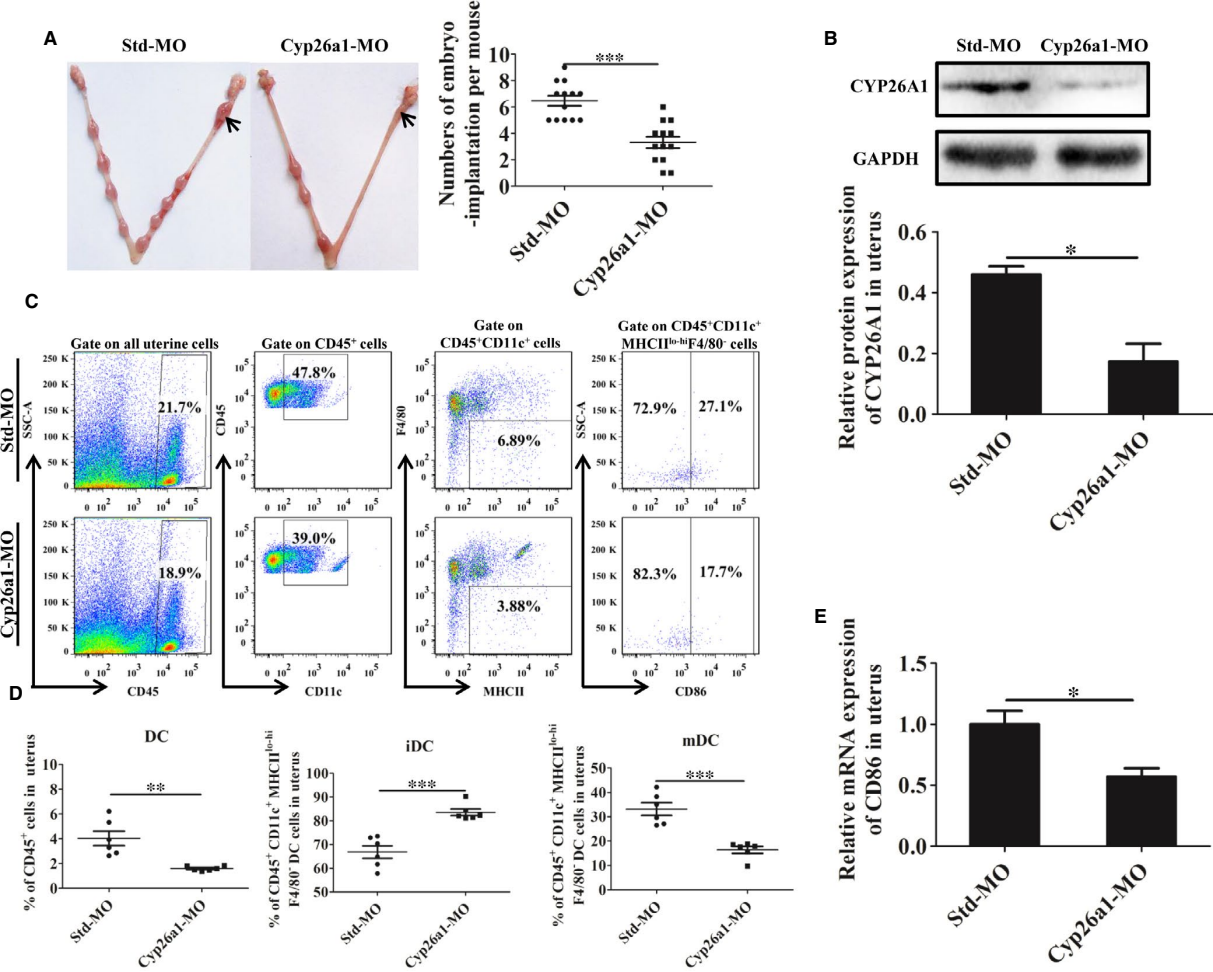
*Cyp26a1*‐MO knockdown mice model. (A), Representational macrophotographs of the uterus injected with MO (left panel) and statistics results of the number of embryo implantation (right panel). Mice were injected with 30nmol Std‐MO or *Cyp26a1*‐MO in the horn of the uterus on the fourth day of pregnancy and sacrificed on the seventh day. Black arrow here represents injection site. The numbers of embryo implanted from 13 mice of each group were counted. (B), Analysis of the expression of CYP26A1 in the uteri by western blot. The data of each group were from three independent biological repeats. (C), One group of representative results of flow analysis. The gating strategy is arranged from the left to right. (D), Statistical analyses of the percentage of dendritic cells in the uterine immune cells and the percentages of immature and mature DC in DCs. The data of each group are presented from six independent biological repeats. (E), The relative expression of CD86 in uterus was analysed by quantitative PCR. The data were obtained from four mice in each group. DC, dendritic cell; iDC, immature dendritic cell; mDC, mature dendritic cell. *Cyp26a1*‐MO, *Cyp26a1* specific morpholino antisense oligonucleotides; Std‐MO, standard control morpholino oligos. *** *P* ≤ 0.001; ** *P* ≤ 0.01; * *P* ≤ 0.05

To investigate the relationship between CYP26A1 and DCs, we constructed a *Cyp26a1* knockdown mouse model by intrauterine injection *Cyp26a1*‐MO. The control group was injected with Std‐MO. Compared with the control group, the number of embryo implantation in the *Cyp26a1*‐MO treatment mice decreased sharply (Figure 2A, *P* < 0.001). The result of western blot showed that the production of CYP26A1 protein significantly reduced in the treated group (Figure 2B, *P* < 0.05). The average relative protein expression of CYP26A1 in control group and treatment group were 0.46 and 0.17 respectively. Therefore, the knockdown efficiency reached about 63%, which confirmed that intrauterine injection the specific *Cyp26a1*‐MO hindered the expression of CYP26A1 protein in uteri.

Knockdown *cyp26a1* significantly altered the proportion of uDCs and their sub‐populations. The proportions of uDCs and their sub‐populations were analysed by flow cytometry (Figure 2C,D). CD45^+^CD11c^+^MHCII^lo‐hi^F4/80^−^ DCs in total uterine immune cells remarkably decreased in *Cyp26a1*‐MO knockdown mice on GD7 (Figure 2D, *P* < 0.01). Among these DCs, the immature DCs increased significantly in the *Cyp26a1*‐MO treated group, whereas mature DCs decreased extremely (Figure 2D, *P* < 0.001). Real‐time PCR showed that the relative mRNA level expression of mature DC marker molecule CD86 also decreased significantly in the *cyp26a1* knockdown mice uteri (Figure 2E, *P* < 0.05). These data suggest that CYP26A1 might be closely related to the number or the differentiation and maturation of DC in mice uterus.

### 3.3 Immunized with pCR3.1‐Cyp26a1 recombinant plasmid significantly affects the percentages of uDCs and their subsets

**Figure 3:**
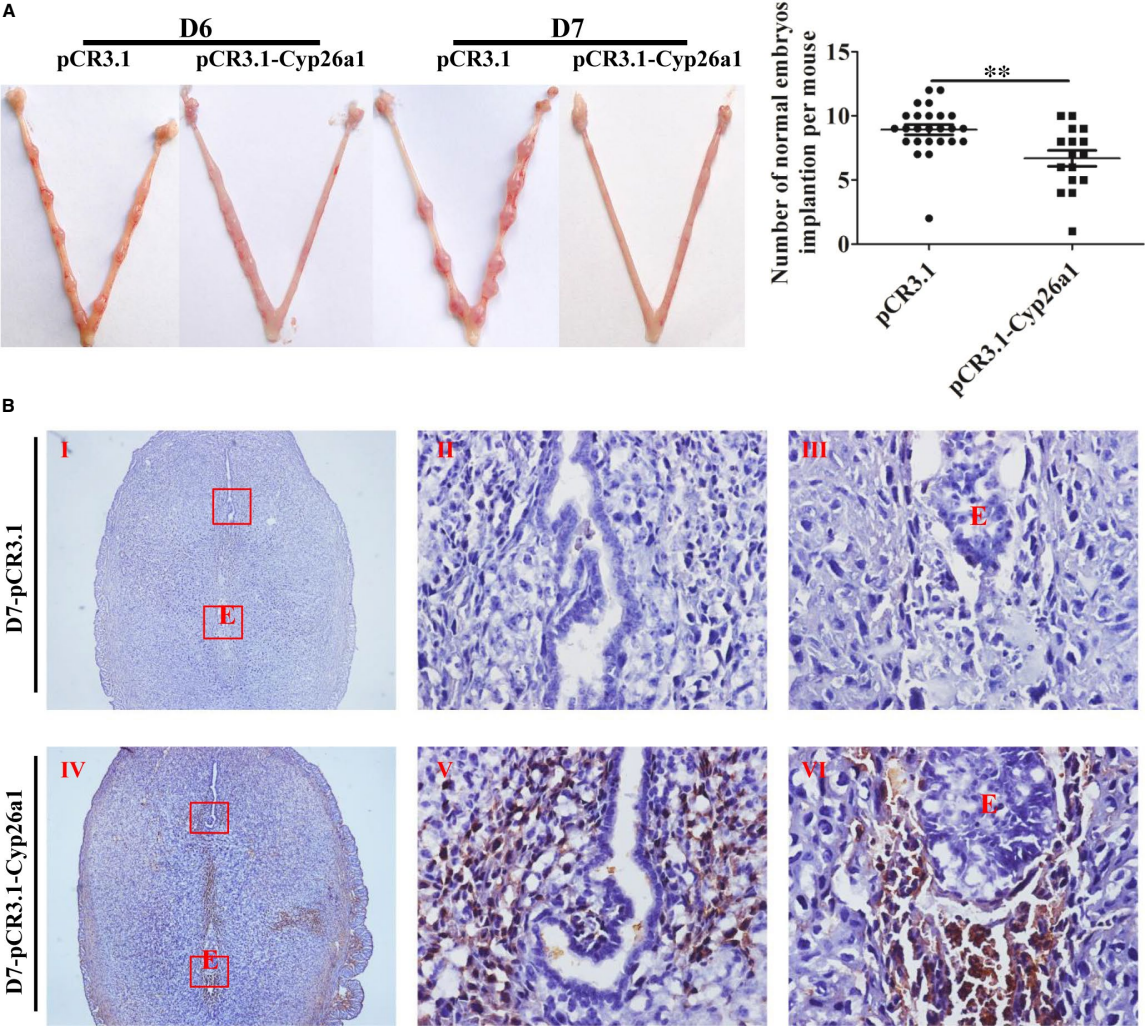
*pCR3.1‐Cyp26a1* recombinant plasmid vaccine immunized mice model. (A), Representational macrophotographs of the uteri on the sixth and seventh days of pregnancy immunized with *pCR3.1‐Cyp26a1* and *pCR3.1* plasmids. The number of normal embryos implanted in each mouse from the gestation day 6 and 7 was counted. (B), Direct immunohistochemical assay to detect the production of anti‐CYP26A1 antibodies in vaccine immunized uteri on gestation day 7. I represents the uteri from the *pCR3.1* immunized mice. II and III represent the higher magnification of the red rectangular box area in panel I. IV represents the uteri from the *pCR3.1‐Cyp26a1* immunized mice. V and VI represent the higher magnification of the red rectangular box area in panel IV. The dark brown signals, those are positive signals in panels IV, V, and VI indicate the production of anti‐CYP26A1 antibodies in *pCR3.1‐Cyp26a1* immunized mice. D6, gestation day 6; D7, gestation day 7; E, Embryo. ** *P* ≤ 0.01

**Figure 4:**
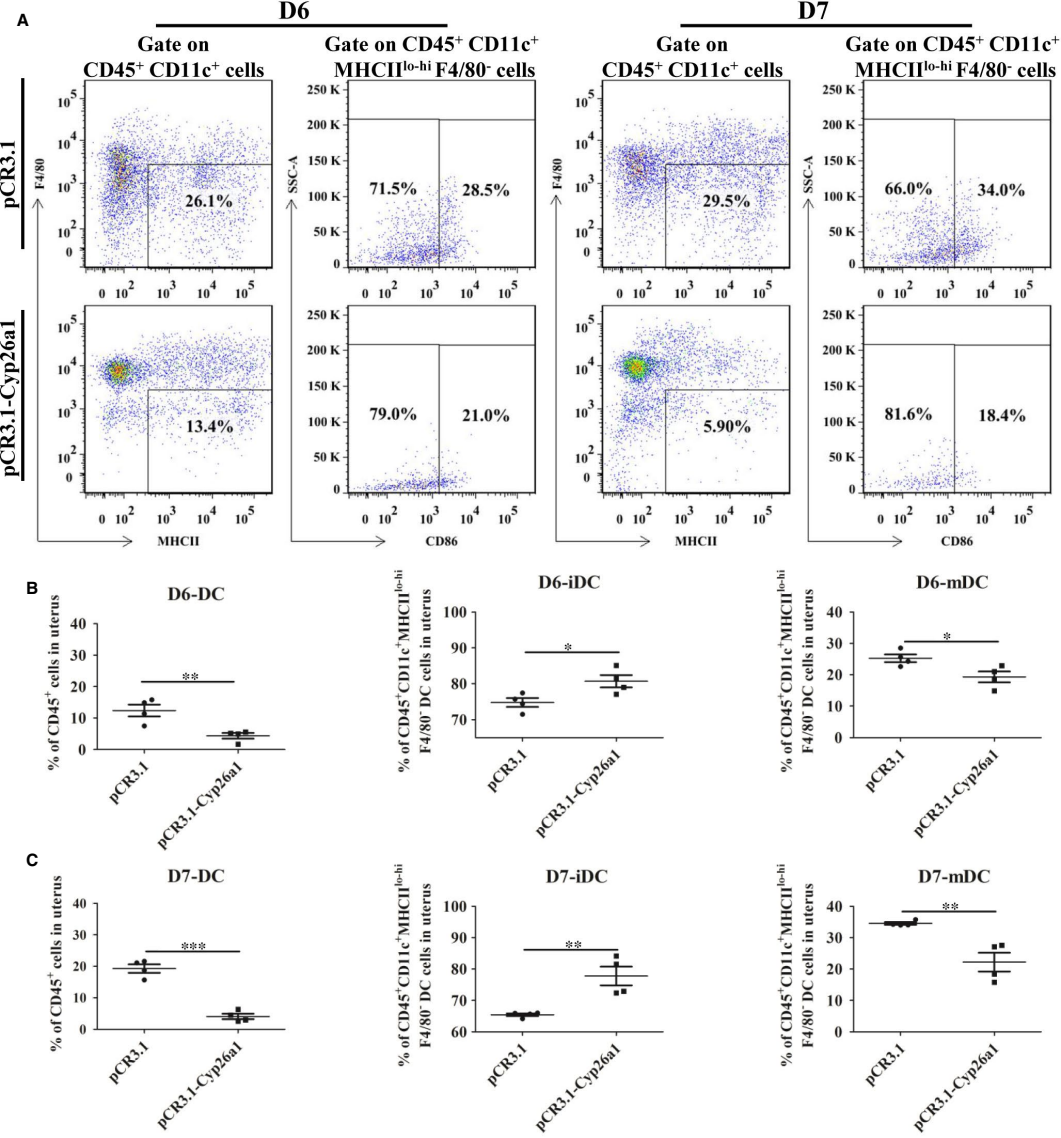
*pCR3.1‐Cyp26a1* vaccine treatment significantly affects the percentage of dendritic cells in uterine immune cells and the percentages of immature and mature dendritic cells in uterine dendritic cells. (A), A representative experiment result of dendritic cells and their subsets from gestation day 6 and 7 was analysed by flow cytometer. The detailed gating strategy is shown in Figure S1. (B), Statistical analysis of the percentage of dendritic cells among the uterine immune cells and the percentages of immature and mature DCs in uterine DCs from the gestation day 6 respectively. The data of each group were from four independent mice. (C) Statistical analysis of the percentage of dendritic cells among the uterine immune cells and the percentages of immature and mature DCs in uterine DCs from the gestation day 7 respectively. The data of each group were from five independent mice. All the data were analysed as using an independent Student's *t* test with graphpad prism software. * *P* ≤ 0.05; ** *P* ≤ 0.01; *** *P* ≤ 0.001. DC, dendritic cell; iDC, immature dendritic cell; mDC, mature dendritic cell. D6, gestation day 6. D7, gestation day 7

To further verify that CYP26A1 might intervene in the percentages of DCs and their subsets, we constructed a vaccine mice model by intramuscular injection of *pCR3.1‐Cyp26a1* recombinant plasmid to produce anti‐CYP26A1 antibodies to depress the function of CYP26A1. Balb/c female mice immunized with *pCR3.1‐Cyp26a1* recombinant plasmid were more difficult to mate with male mice than those immunized with the empty *pCR3.1* plasmid. Most of the *pCR3.1‐Cyp26a1* treated mice had a vaginal plug only after they had been caged with male mice several times. The macroscopic photographs of the uteri from GD6 and GD7 showed that the number of normal implantation embryos significantly decreased in the treated group compared with the control group (Figure 3A, *P* < 0.01). In order to verify the validity of the vaccine model, we performed direct immunohistochemical assay to detect whether antibodies were produced in *pCR3.1‐Cyp26a1* plasmid immunized mice. As shown in Figure 3B, the positive signals were only detected in uteri sections of the treatment group, which indicated that mice immunized with *pCR3.1‐Cyp26a1* produced anti‐CYP26A1 antibodies. The percentages of uDCs and their subsets from GD6 and GD7 were analysed by flow cytometric (Figure 4). The detailed gate strategies were showed in Figure S1. Immunized with *pCR3.1‐Cyp26a1* significantly affected the amount of DCs and their subsets during the peri‐implantation stages. Compared with the control group, uterine CD11c^+^MHC^lo‐hi^F4/80^−^ DCs from the *pCR3.1‐Cyp26a1* vaccine treated group significantly down‐regulated on GD6 (Figure 4A,B, *P* < 0.01) and GD7 (Figure 4A,C, *P* < 0.001). Their subsets iDCs displayed up‐regulation and mDCs showed down‐regulation in *pCR3.1‐Cyp26a1* plasmid immunized uteri both on GD6 (*P* < 0.05) and GD7 (*P* < 0.01). The expression of CD86 decreased significantly in the *pCR3.1‐Cyp26a1* plasmid treated mice uteri both on GD6 and GD7 (*P* < 0.01 and *P* < 0.05 respectively, Figure S2A). These outcomes were similar to our *cyp26a1*‐MO knockdown model, which indicated that CYP26A1 might regulate the percentages of uDCs and their subsets.

### 3.4 Cyp26A1 might regulate the differentiation and maturation of DCs through ID2 and CD86

**Figure 5:**
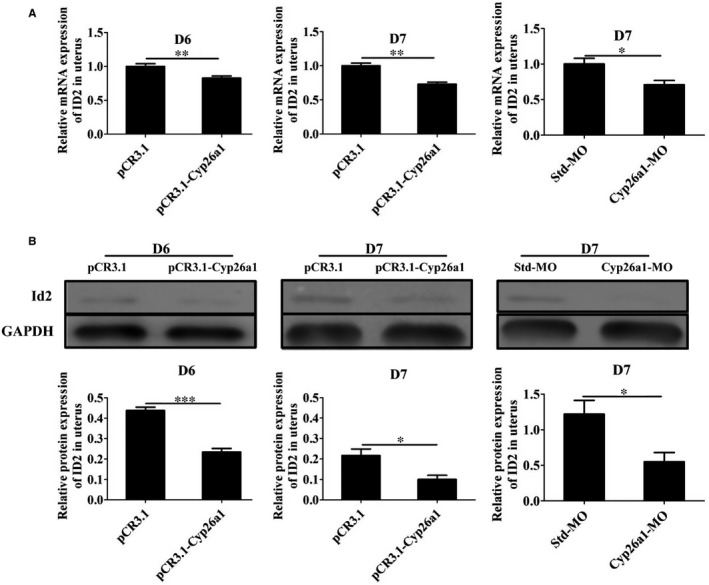
The expression of ID2 in *pCR3.1‐Cyp26a1* plasmid immunized and *Cyp26a1*‐MO knockdown mice models. (A), The relative mRNA level expression in uteri from these two mice models were analysed by real‐time PCR. (B), The protein expression in the uteri from the two mice models were analysed by western blot. All the data were obtained from four biological repeats in each group. D6, gestation day 6; D7, gestation day 7; *Cyp26a1*‐MO, *Cyp26a1*‐specific morpholino antisense oligonucleotides; Std‐MO, standard control morpholino oligos; ID2, Inhibitor of DNA‐binding protein 2. * *P* ≤ 0.05; ** *P* ≤ 0.01; *** *P* ≤ 0.001

In order to explore the potential regulatory mechanism of CYP26A1 on uDCs, we first performed the transcriptome sequencing analysis of iDCs and mDCs from normal pregnant mice uteri without any treatment. Some transcription factors related to DC development and markers related to DC maturation showed significant differences between iDCs and mDCs (Table 1). The higher expression of *Cd86* and *Cd83*, and the lower expression of *Ly6C* in mDCs than those in iDCs indicated the reliability of these two cell subsets sorted by flow cytometry. Transcription factors *Id2*, *Irf8*, *Irf4,* *Runx2* and *Spib* have been reported to be involved in the development of DCs.^30^, ^31^, ^32^, ^33^ *Id2* and *Irf8* significantly up‐regulated in mDCs, whereas *Irf4,* *Runx2* and *Spib* significantly down‐regulated in mDCs (Table 1). In order to explore whether CYP26A1 affects the differentiation and maturation of DCs through these genes, we performed real‐time PCR and western blot experiments. Real‐time PCR showed that the expression of ID2 significantly decreased in both *pCR3.1‐Cyp26a1* treated and *Cyp26a1*‐MO treated mice models (Figure 5A), while the expression of RUNX2*,* SPIB*,* IRF4 and IRF8 was not significantly different in these two models (data were not displayed). Western blot further proved the protein level expression of ID2 significantly reduced in these two treated mice uteri (Figure 5B). To prove that CYP26A1 participated in the development of DCs, we carried out a bone BMDCs induced experiment in vitro. The expression of ID2 also significantly decreased in BMDCs under *Cyp26a1*‐MO treatment (Figure S2B). Meanwhile, CD86 also displayed a significant down‐regulation in the *Cyp26a1*‐MO treated BMDCs (Figure S2C). These results suggest that CYP26A1 might regulate the differentiation and maturation of DCs through ID2 and CD86.

**Table 1:** Some transcription factors related to DC development and markers related to DC maturation were obviously different between mice uterine immature and mature dendritic cells under regular rearing condition

Gene_id	Gene name	iDC_FPKM	mDC_FPKM	log2FC(mDC/iDC)	Up‐down‐regulation (mDC/iDC)
ENSMUSG00000020644	Id2	555.01	1770.14	1.67	Up
ENSMUSG00000021356	Irf4	10.32	2.49	−2.01	Down
ENSMUSG00000041515	Irf8	118.35	379.17	1.68	Up
ENSMUSG00000039153	Runx2	6.74	1.11	−2.5	Down
ENSMUSG00000008193	Spib	13.57	0.81	−3.91	Down
ENSMUSG00000022901	CD86	20.9	72.16	1.78	Up
ENSMUSG00000015396	CD83	430.51	1250.37	1.54	Up
ENSMUSG00000079018	Ly6c1	163.09	69.36	−1.23	Down
ENSMUSG00000022584	Ly6c2	74.2	28.39	−1.38	Down

Abbreviations: FPKM, Fragments Per Kilobase of transcript sequence per Millions base pairs sequenced; log2FC(mDC/iDC), the log to base 2 of the difference in transcript abundance between the immature and mature dendritic cells; iDC, immature dendritic cell; mDC, mature dendritic cells.

## 4 DISCUSSION

**Figure 6:**
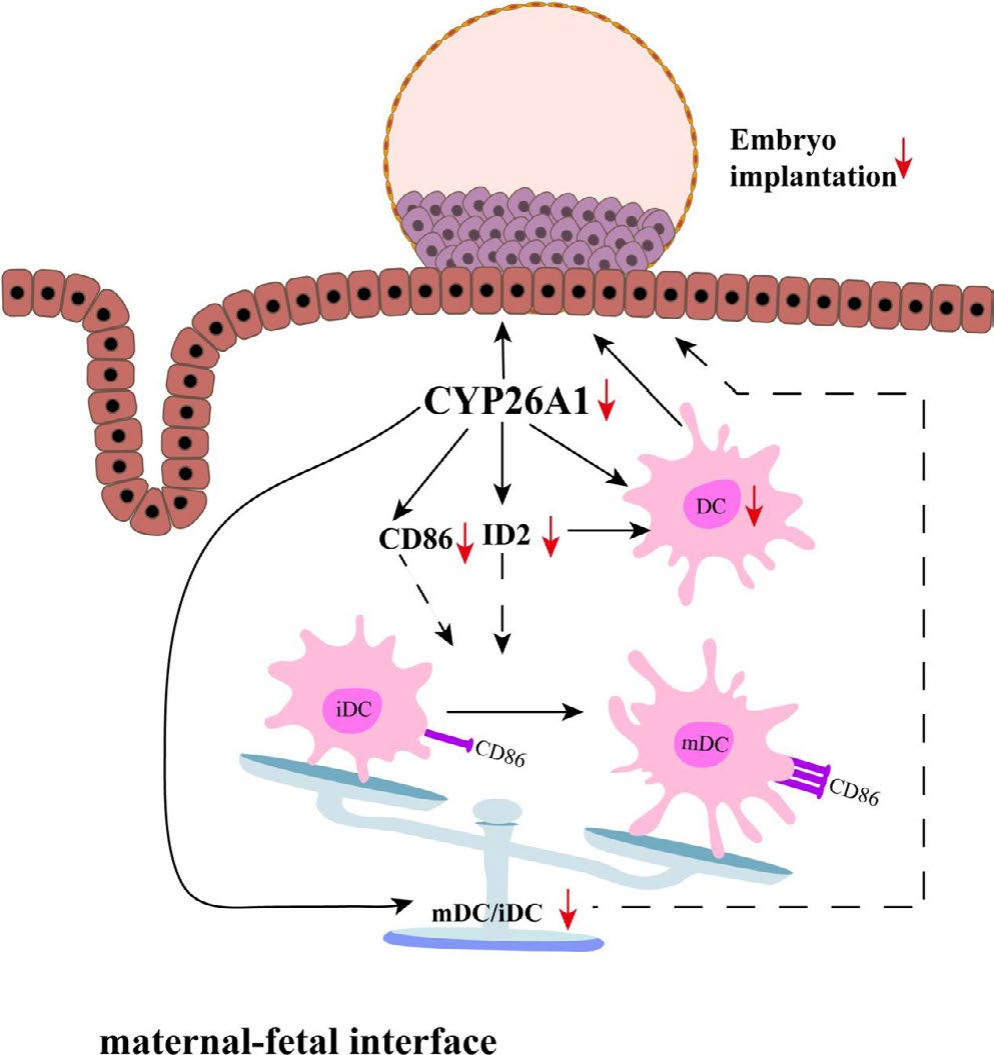
Circuit diagram of CYP26A1 affecting embryo implantation through regulating dendritic cells. Inhibition the expression or function of CYP26A1 led to the reduction in the expression of CD86, ID2, the percentage of dendritic cells and the ratio of mature and immature DC in uteri. The decrease in dendritic cell impacted the embryo implantation. The reduction in the ratio between mature and immature DCs might also affect embryo implantation. Black arrows represent promotion. Black false arrow represents a hypothetical result has not yet been confirmed. The red downward arrow means down‐regulation. CYP26A1, Cytochrome P450 26A1; DC, dendritic cell; iDC, immature dendritic cell; mDC, mature dendritic cell; ID2, Inhibitor of DNA‐binding protein 2

In this study, we mainly explored whether CYP26A1 involved in embryo implantation is related to DCs. Inhibition the expression or function of CYP26A1 decreased the number of uterine DCs, influenced the original balance between uterine iDCs and mDCs and reduced the expression of ID2 and CD86, which suggest that CYP26A1 may regulate the differentiation of uterine DCs by ID2 and CD86 to participate in embryo implantation (Figure 6). As far as we know, this is the first study to correlate CYP26A1 with DCs both in vivo and in vitro.

*Cyp26a1*‐MO knockdown mice model and *pCR3.1‐Cyp26a1* plasmid immunized mice model were used. Antisense oligonucleotides are sequence‐specific binding polymers designed to block protein translation of target messenger RNA. Morpholinos can afford antisense oligos with many merits such as very high efficacy and specificity, immunity to nucleases and good aqueous solubility.^34^ It has been reported that morpholinos can effectively disrupt protein expression by penetrating mouse uterine luminal epithelium and the underlying stromal cells after intrauterine injection.^5^, ^8^, ^25^ So we injected *Cyp26a1*‐MO directly into the uterine cavity using intrauterine injection technique and obtained similar results as previously reported, such as the decrease in the production of CYP26A1 protein and the number of embryo implantation in treatment group.^5^, ^8^ *pCR3.1‐Cyp26a1* recombinant plasmids immunized model also has many advantages such as low cost and easy acquisition. Plasmid immunization could directly transfect animal cells in vivo and induces a long‐term antibody response.^35^, ^36^ Our laboratory has successfully constructed a *pCR3.1‐Cyp26a1* recombinant plasmid that could express CYP26A1 antibody in mice.^5^, ^7^, ^8^, ^26^ In this study, we also used this model to explore the relationship between CYP26A1 and DCs and again verified its feasibility.

The percentage of uterine CD11c^+^MHCII^lo‐hi^F4/80^−^ DCs was significantly decreased in *Cyp26a1*‐MO‐treated and *pCR3.1‐Cyp26a1*‐immunized mice, as compared to their corresponding controls (Figures 2D and 4B,C). Dendritic cell has been well known to play vital roles in reproductive process. It has been reported that depletion of DCs during implantation process impaired the implantation and early placental development, resulting in a reduced breeding efficiency.^10^, ^14^, ^15^ Therefore, the reduction in uterine DCs in this study is considered to be one of the most important reasons for the decrease of embryo implantation.

On the whole, the proportion of uterine immature DCs was much higher than that of mature DCs in both treated and control groups (Figures 1B, 2D and 4B,C). Immature DCs tend to mediate immune tolerance, whereas mature DCs tend to mediate immune rejection. It has been reported that DCs could regulate the Th1/Th2 balance to maintain a Th2‐dominant state in early human pregnancy decidua.^37^ Thus, maintain a higher proportion of immature DCs during early pregnancy are necessary. As shown in Figures 2A and 3A, normal embryo implantation sites also existed in the treatment groups.

The blastocyst was eventually successfully implanted into the acceptable endometrium through apposition, adhesion and infiltration, which requires strong inflammatory response.^38^, ^39^ Immune cells such as NK cells, DCs and T cells play key roles in this process. IDCs mainly promote T‐cell tolerance, whereas mDCs mainly induce T‐cell immunity and generate proinflammatory responses.^40^ In this study, uterine mature DCs increased from GD5 to GD7 in normal pregnant mice under regular feeding conditions without any treatment (Figure 1B). However, compared with the control groups, the number of mature DCs was significantly lower and the number of immature DCs was significantly higher in the uteri on GD6 and GD7 from the treatment groups in both *Cyp26a1*‐MO knockdown and *pCR3.1‐Cyp26a1* vaccine mice models (Figures 2D, 4B,C). Similar with our previous report, blocking the function of CYP26A1 reduced uterine Th17 cells (mediate immune rejection) during the peri‐implantation.^7^ These phenomena may dampen inflammatory processes and impact embryo implantation.

Well, how does CYP26A1 regulate the number of DCs? Whether CYP26A1 is related to the differentiation and maturation of DCs? We found the expression of ID2 and CD86 significantly decreased in the uteri with *Cyp26a1*‐MO and *pCR3.1‐Cyp26a1* vaccine treatment groups (Figures 2E, 5 and 6 and Figure S2). It has been reported that ID2 is up‐regulated during DC development in vitro and crucial for the development of distinct DC subsets in vivo.^30^, ^41^, ^42^ *Id2* ^−/−^ mice lack Langerhans cells, the cutaneous contingent of DCs and the splenic CD8α^+^ DC subsets.^30^ CD86 is a well‐known DC mature maker. Our RNA sequencing data of iDCs and mDCs also showed ID2 and CD86 were up‐regulated in mDCs (Table 1). Therefore, the decreased expression of ID2 and CD86 might directly reduce the proportion of DCs, especially mDCs in vivo. We also found that the expression of ID2 and CD86 decreased in BMDC (Here, the cells included a small number of other antigen‐presenting cells) treated with *Cyp26a1*‐MO in vitro (Figure S2). These data suggest that CYP26A1 affects the differentiation and maturation of DCs by regulating the expression of ID2 and CD86 (Figure 6). We know that hormones such as progesterone and E2 could regulate the differentiation and/or maturation of DCs.^19^, ^43^ However, whether CYP26A1 regulates the differentiation and maturation of DCs is related to hormone has not been seen the relevant reports. Except the differentiation and maturation of DCs, their endocytosis and migration are also indispensable components in immune regulation. A major function of DCs is their capacity of endocytosis, which can initiate immune response by capturing and processing antigens, expressing MHCII and costimlatory molecules. In addition, DCs could produce cytokines and chemokines to attract DCs and NK cells to inflammation and initial infection sites.^44^ It remains unclear that whether blocking CYP26A1 affects the endocytosis, migration and chemokine secretion of DCs.

In summary, our data suggest that blocking the expression/function of CYP26A1 leads to the decrease in DCs, especially the number of mature DCs in the uteri of mice during peri‐implantation, which might be one of the important reasons for the decrease in embryo implantation. This is a novel regulatory mechanism and the first evidence that CYP26A1, a metabolic enzyme, affects the immune response during peri‐implantation via modulating DCs.

## CONFLICT OF INTEREST

The authors confirm that there are no conflicts of interest.

## AUTHOR CONTRIBUTION

J‐PP designed the experiments, provided critical reagents and experimental expertise and supervised the study; A‐QG designed the experiments, performed the experiments, analysed the data and wrote the manuscript; D‐DL, D‐PW, Y‐QL, W‐HJ, and YY performed some of the experiments; H‐YL analysed some data.

## Data Availability

We confirm that the data in our paper can be used.
