# Supplementary figures and images for: Cytochrome P450 26A1 modulates uterine dendritic cells in mice early pregnancy
Source: J Cell Mol Med. 2019 May 31;23(8):5403–14. doi: 10.1111/jcmm.14423 (PMC6652875; doi:10.1111/jcmm.14423)

Supplementary Fig. 1

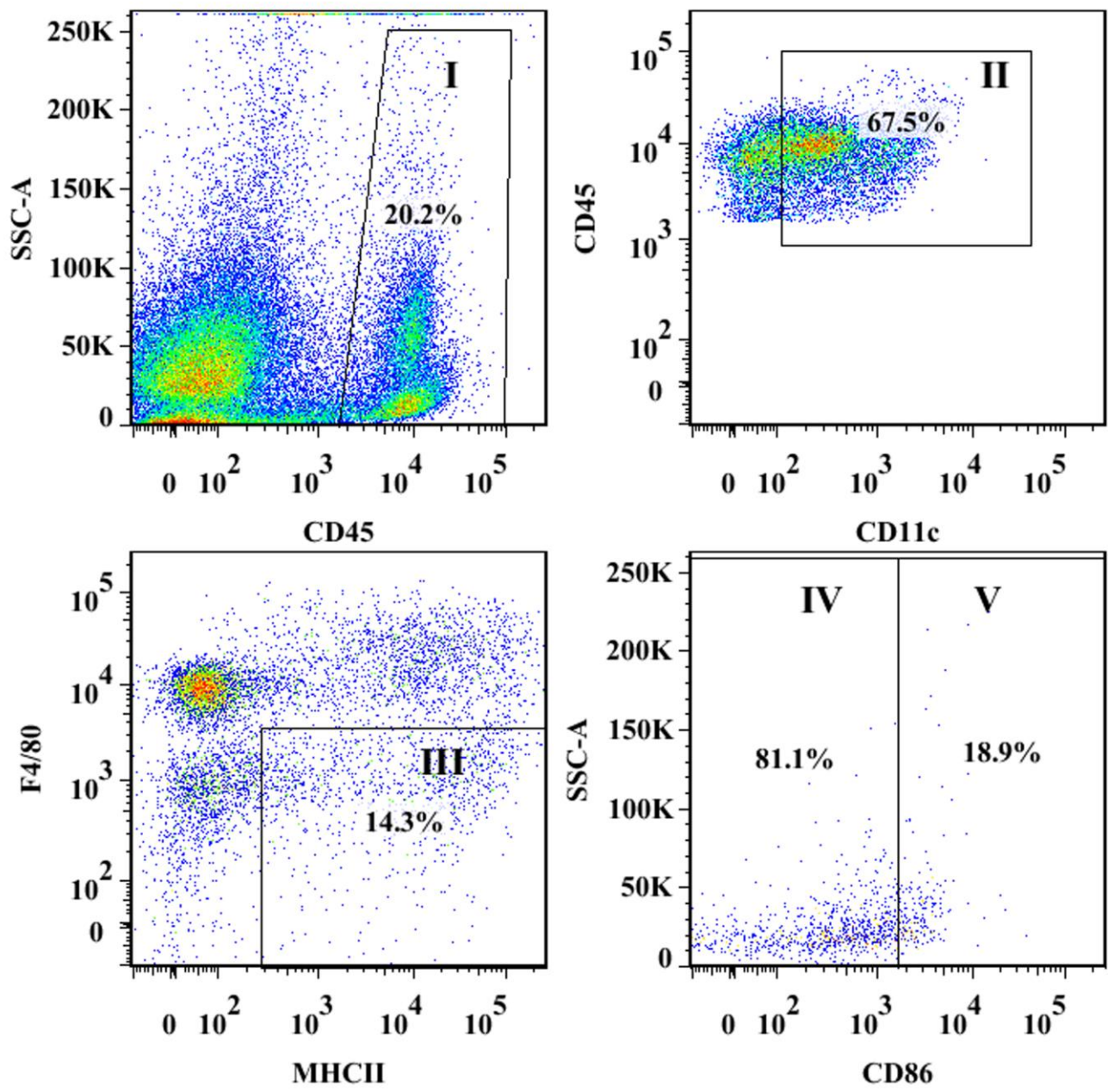

Supplementary Fig. 2

A

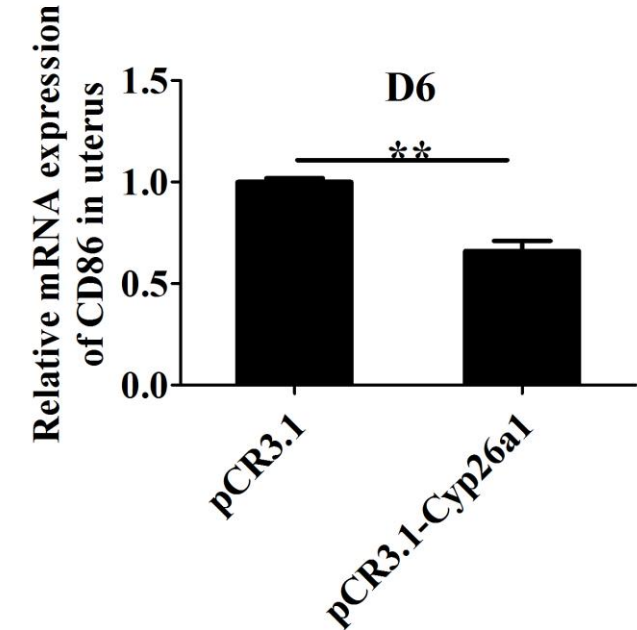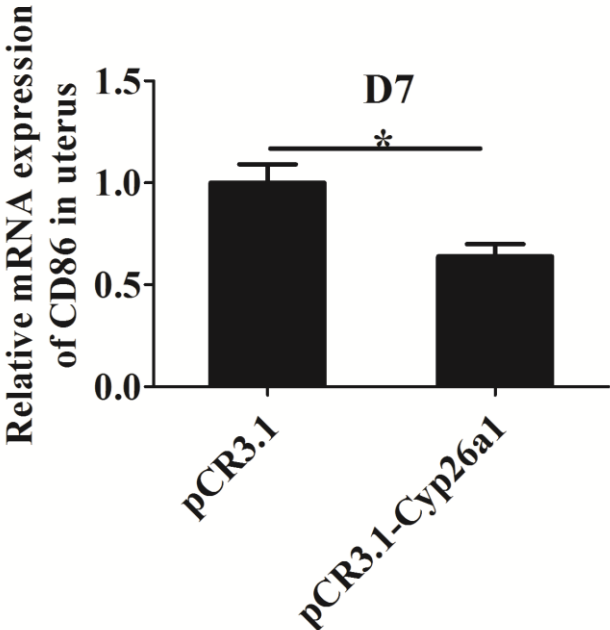

B

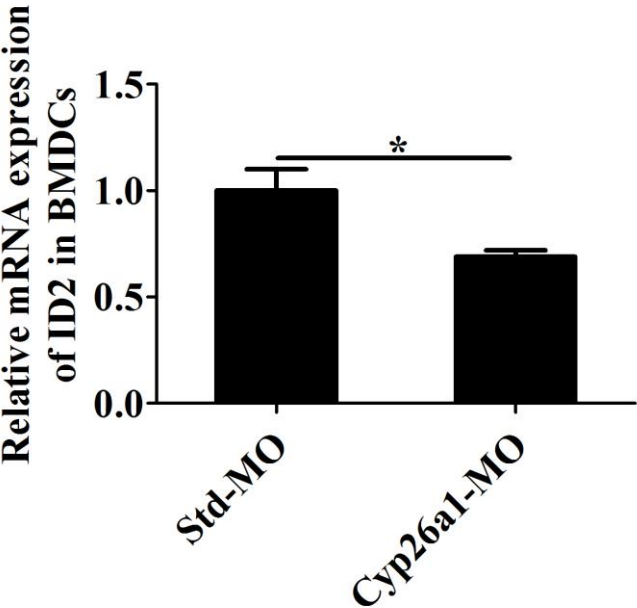

C

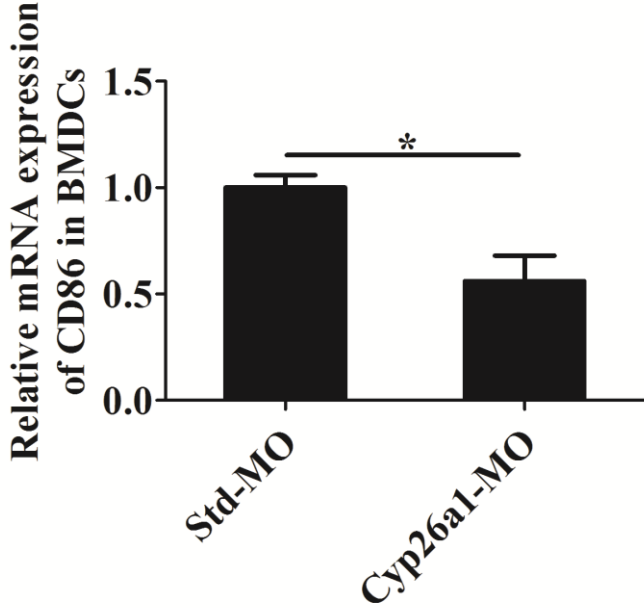

Supplement: Supplementary file 1 [file JCMM-23-5403-s001.pdf]
